# The genetic basis of classical galactosaemia in Polish patients

**DOI:** 10.1186/s13023-021-01869-3

**Published:** 2021-05-24

**Authors:** Aleksandra Jezela-Stanek, Anna Bauer, Katarzyna Wertheim-Tysarowska, Jerzy Bal, Agnieszka Magdalena Rygiel, Jolanta Sykut-Cegielska

**Affiliations:** 1 Department of Genetics and Clinical Immunology, National Institute of Tuberculosis and Lung Diseases, Warsaw, Poland; 2 Department of Inborn Errors of Metabolism and Paediatrics, Institute of Mother and Child, Warsaw, Poland; 3 Department of Medical Genetics, Institute of Mother and Child, Warsaw, Poland

**Keywords:** *GALT* gene, Variants, Classical galactosemia

## Abstract

Classic galactosemia (OMIM #230400) is an autosomal recessive disorder caused by homozygous or compound heterozygous pathogenic variants in the galactose-1-phosphate uridylyltransferase gene (*GALT*; 606999) on chromosome 9p13. Its diagnosis is established by detecting elevated erythrocyte galactose-1-phosphate concentration, reduced erythrocyte galactose-1-phosphate uridylyltransferase (GALT) enzyme activity. Biallelic pathogenic variants in the *GALT* gene is confirmed by DNA analysis. Our paper presents molecular characteristics of 195 Polish patients diagnosed with galactosemia I, intending to expand the current knowledge of this rare disease's molecular etiology. To the best of our knowledge, the described cohort of galactosemia patients is the largest single-center cohort presented so far.

**Supplementary Information:**

The online version contains supplementary material available at 10.1186/s13023-021-01869-3.

## Introduction

Classic galactosemia (OMIM: Galactosemia I, GALAC1, Galactosemia, Duarte variant, included, #230400) is an autosomal recessive disorder caused by biallelic pathogenic variants in the galactose-1-phosphate uridylyltransferase coding gene (*GALT*; 606999) on chromosome 9p13. However, mutations in other genes (*GALK, GALE*) involved in Leloir's pathway of galactose metabolism may also occur, resulting in galactosemia II (GALAC2; 230200) and galactosemia III (GALAC3; 230350), respectively. Moreover, in 2019 galactosemia IV (GALAC4; 618881), caused by homozygous or compound heterozygous state in the *GALM* gene, was described [gene encodes the enzyme galactose mutarotase (EC 5.1.3.3), which converts beta-D-galactose to alpha-D-galactose, the first step in the Leloir's pathway] [1].

*GALT* defects lead to two types of galactosemia: the classic and biochemical variant ones (often referred to as Duarte galactosemia). The classic form usually manifests in the neonatal period, after galactose ingestion, with jaundice, hypoglycemia, liver insufficiency, vomiting and diarrhoea, renal tubular dysfunction, muscle hypotonia, cataract and urosepsis. Long-term complications, may include intellectual disability, verbal dyspraxia, motor abnormalities, and hypergonadotropic hypogonadism [2]. Early diagnosis, followed by treatment with a lactose-restricted (dairy-free) diet, is undeniably essential to avoid health issues, but some complications may still occur [3]. On the contrary, in Duarte galactosemia, where enzyme activity is typically reduced by 75%, the symptoms are much milder and dietary intervention is usually unnecessary [4]. On the molecular level, the classic galactosemia is caused by biallelic pathogenic variants, while in Duarte galactosemia, the so-called Duarte 2 variant (c.[-119_-116delGTCA;940A>G]) is either homozygous or heterozygous with pathogenic *GALT* variant [5]. Within the symptomatic spectrum of galactosemia, clinical variant galactosemia is also known. It occurs in African Americans, and native Africans in South Africa thus is not covered within our article [Berry 2000].

The prevalence of classic galactosemia depends on ancestry. This disease is more common among individuals of European ancestry than in the Asia population. Its prevalence in the UK ranges from 1:23,500 to 1:44,000, while it differs from 1:50,000 in the US to 1:400,000 in Taiwan and 1:700,000 in Japan [6, 7]. In Poland, the prevalence of "classical galactosemia" is estimated at 1:35,000 [8, 9].

In many countries, due to treatment options, hereditary galactosemia is a part of the Newborn Screening (NBS) programs [10]. However, GALAC1 is not included in Poland's newborn NBS program; therefore, most of the patients are diagnosed due to severe life-threatening symptoms, followed by an enzymatic test and molecular studies and/or positive family history. Only in the West Pomeranian region of the country, NBS for GALAC1 has been performed within a cross-border cooperation program.

The study aimed to summarize the results of genotyping of a Polish cohort of patients from a single centre—Institute of Mother and Child (who serves as an unofficial national reference centre for the patients with galactosemia), to get further insight into molecular defects in the *GALT* gene and to present the phenotypic variability on the examples of two families with novel *GALT* variants.

## Patients and methods

### General characteristics of reported patients

One hundred ninety-five patients from 175 families (hence 175 probands and 20 siblings) born between 1972 and 2020 were included in the study based on clinical/biochemical galactosemia suspicion and/or positive family history. As many as 190/195 (97%) patients from this group are treated in a single reference centre (Department of Inborn Errors of Metabolism and Paediatrics, Institute of Mother and Child in Warsaw).

In the neonatal period, the probands often presented cachexia, hyperbilirubinemia, coagulation disorders, tubulopathy, generalized oedema or cataract. Sepsis, urinary tract infections, or meningitis also occurred in many cases. The younger siblings were tested on day 1 of their lives and received a lactose-free formula from the first feeding. As a result, if confirmed, they were diagnosed in the asymptomatic period. Besides, one patient born in Ireland was diagnosed with NBS there; in four families, the proband was the younger child, and the diagnosis of older siblings was made with a delay.

Despite dietary treatment, many patients develop long-term complications. We often observed cognitive problems, intellectual disability, defects in speech and language development. Some patients complain of hand tremors. Primary ovarian failure is seen in many women with galactosemia. A significant problem in these patients is infertility, although five women from our group gave birth to healthy offspring. Diminished bone mineral density is not common thanks to the supplementation of calcium and vitamin D3.

### History of individuals in whom novel *GALT *variants have been identified

#### Family A

The first child with atypical classic galactosemia was diagnosed at the age of 5 months. Currently, a 5-year-old girl is the first child of unrelated, healthy parents. She was born after the uncomplicated pregnancy and delivery. She was breastfed, but due to her weight loss of up to 10% on the third day of life, urine analysis was ordered and revealed slight transient proteinuria from 1 month (0.4–1.85 g/l; normal values < 0.12 g/l). Urinary tract infection was excluded, and then her weight gain was adequate. The ultrasound examination of the abdominal organs demonstrated no abnormalities. Initially, the liver enzymes were in the normal range, but at 2 months of age, slight hypertransaminemia was observed (ALT 105 U/L, AST 165 U/L; normal values 7–40, 20–72, respectively). No coagulopathy was found. Two months later, her parents noticed nystagmus. An ophthalmological examination showed bilateral cataracts.

Further investigations revealed a significantly reduced GALT activity (5%; 0.12 mmol UDPgal/h/RBC; normal values 1.81–2.57) which confirmed the diagnosis of classic galactosemia. A lactose-free diet was introduced at the age of 5 months, and then proteinuria and hypertransaminasemia resolved. The girl underwent cataract surgery and intraocular lens implantation.

The proband's younger sister had an enzyme analysis on the first day of her life. A significant decrease in GALT activity was found (1%; 0.02 mmol UDPgal/h/RBC; normal values 1.46–2.21). The child was fed a lactose-free formula from the beginning and showed no clinical symptoms.

The girls are currently 5 and 3 years old. Their intellectual and speech development is as expected. Due to their still young age, the occurrence of long-term galactosemia complications cannot be assessed yet.

#### Family B

A boy was born at 40 weeks of gestation after an uneventful delivery. After birth, when breastfed, he developed severe jaundice from the second day of life (with a total bilirubin of 23.4 mg/dl), cholestasis, hypertransaminasemia and coagulation disorders. Moreover, urinary tract infection and *E. coli* sepsis were found. On day 5 of life, enzymatic research revealed undetectable GALT activity confirming a diagnosis of galactosemia. The diet was changed to lactose-free, with gradual clinical improvement and symptom relief.

Despite a properly followed diet, the boy had long-term galactosemia complications such as delayed speech development, impaired sensory integration, learning difficulties, and disharmonious intellectual development.

The boy had severe symptoms in newborn age and then long-term complications of galactosemia, and his enzyme activity was undetectable.

### Molecular methods

DNA was isolated from peripheral blood leukocytes. The molecular test was perform using Sanger sequencing. The analysis procedure involved a two-step approach—first, analysis of exons 6–9, and, if the genotype was determined, the remaining ones in the second step. The PCR and sequencing primers were self-designed (Additional file 1: Table 1). Fluorograms were analyzed using Mutation Surveyor Software (Softgenetics) and evaluate using HGMD (Human Gene Mutation Database, http://www.hgmd.cf.ac.uk), *GALT* database [11], and ACMG (American College of Medical Genetics and Genomics) classification [12] using tools available at the VARSOME website (https://varsome.com). The sequence NM_000155.4 was used as a reference.

## Results

### Molecular characteristics of diagnosed individuals

#### Novel variants

The p.Pro87Leu is not registered in any database, while the p.Glu352Gln has only been reported once in GnomAD (per 251,424 tested alleles) and is not present in any clinical resource. Both variants are assigned as likely pathogenic using ACMG criteria, and bioinformatic evaluation on the VARSOME platform showed that, in both cases, 19 out of 21 pathogenicity ranging algorithms classifies them as damaging. The p.Pro87Leu was detected *in trans* with a known pathogenic variants p.Tyr209Ser in patients from Family A, while p.Glu352Gln was *in trans* with p.Gln188Arg in patients from Family B.

#### Characteristics of the studied cohort

Among 175 probands, the full genotype was determined in 173 cases (99%). In 2 remaining patients with a mutation in one allele of *GALT*, only checking exons 6–9 was performed. In total, we identified 29 different mutations in the *GALT* gene on 348 of 350 alleles, including two novel variants: p.Pro87Leu and p.Glu352Gln (Tables 1, 2).

**Table 1:** *GALT* variants detected in the Polish cohort

Mutation		Alleles		HGMD
Coding sequence	Protein level	No	Frequency (%)	
c.563A>G	p.Gln188Arg	172	49.4	CM910169
c.855G>T	p.Lys285Asn	112	32.2	CM920296
c.329-2A>C	NA	11	3.2	CS951420
c.997C>T	p.Arg333Trp	6	1.7	CM910170
c.626A>C	p.Tyr209Ser	6	1.7	CM990655
c.958G>A	p.Ala320Thr	4	1.1	CM950557
c.152G>T	p.Arg51Leu	4	1.1	CM990628
c.425T>A	p.Met142Lys	4	1.1	CM910168
c.-119_-116delGTCA;c.940A>G	NA;p.Asn314Asp	3	0.9	CD991729 + CM940804
c.812A>G	p.Glu271Gly	2	0.6	CM074209
c.1014C>G	p.Gly338Gly	2	0.6	CS129678
c.584T>C	p.Leu195Pro	2	0.6	CM920295
c.1138T>C	p.Ter380Arg	2	0.6	CM990679
c.499T>C	p.Trp167Arg	2	0.6	CM990646
c.626A>G	p.Tyr209Cys	2	0.6	CM990656
c.507+2T>C	unknown	1	0.3	CS991403
c.83-11>G	unknown	1	0.3	CS129673
c.611G>C	p.Arg204Pro	1	0.3	CM990653
c.982C>T	p.Arg328Cys	1	0.3	CM110770
c.152G>A	p.Arg51Gln	1	0.3	CM051924
c.490C>T	p.Gln164Ter	1	0.3	CM990645
c.505C>A	p.Gln169Lys	1	0.3	CM993449
c.1054G>C	p.Glu352Gln	1	0.3	novel
c.392T>G	p.Phe131Cys	1	0.3	CM074218
c.285T>G	p.Phe95Leu	1	0.3	CM994053
c.748C>A	p.Pro250Thr	1	0.3	CM074211
c.260C>T	p.Pro87Leu	1	0.3	novel
c.404C>T	p.Ser135Leu	1	0.3	CM950546
c.134C>T	p.Ser45Leu	1	0.3	CM990627

*NA* doesn't apply, *unknown* not detected

**Table 2:** *GALT* genotypes in the Polish cohort

Genotype						Families		Patients	
ALLEL 1			ALLEL 2			n	%	n	%
cDNA name	Amino acid name	Effect	cDNA name	Amino acid name	Effect				
c.563A>G	p.Gln188Arg	m	c.563A>G	p.Gln188Arg	m	53*	30	60	31
c.563A>G	p.Gln188Arg	m	c.855G>T	p.Lys285Asn	m	39	22	41	21
c.855G>T	p.Lys285Asn	m	c.855G>T	p.Lys285Asn	m	26*	15	29	15
c.563A>G	p.Gln188Arg	m	c.997C>T	p.Arg333Trp	m	3	2	4	2
c.855G>T	p.Lys285Asn	m	c.958G>A	p.Ala320Thr	m	3	2	3	2
c.855G>T	p.Lys285Asn	m	c.997C>T	p.Arg333Trp	m	3	2	3	2
c.152G>T	p.Arg51Leu	m	c.563A>G	p.Gln188Arg	m	3	2	4	2
c.626A>G	p.Tyr209Cys	m	c.563A>G	p.Gln188Arg	m	2	1	3	2
c.855G>T	p.Lys285Asn	m	c.1138T>C	p.Ter380Arg	STOP codon loss	2	1	3	2
c.855G>T	p.Lys285Asn	m	c.83-11T>G		spl	1	1	2	1
c.563A>G	p.Gln188Arg	m	c.-119_-116delGTCA; c.940A>G	NA;p.Asn314Asp	spl	2	1	2	1
c.329-2A>C	nd	spl	c.563A>G	p.Gln188Arg	m	1	1	2	1
c.563A>G	p.Gln188Arg	m	c.1014C>G	p.Gly338Gly	spl	2	1	2	1
c.329-2A>C	nd	spl	c.855G>T	p.Lys285Asn	m	2	1	2	1
c.425T>A	p.Met142Lys	m	c.855G>T	p.Lys285Asn	m	2	1	2	1
c.563A>G	p.Gln188Arg	m	c.425T>A	p.Met142Lys	m	1	1	2	1
c.260C>T	p.Pro87Leu	m	c.626A>C	p.Tyr209Ser	m	1	1	2	1
c.563A>G	p.Gln188Arg	m	c.329-2A>C	NA	spl	1	1	1	1
c.855G>T	p.Lys285Asn	m	c.329-2A>C	NA	spl	2	1	2	1
c.855G>T	p.Lys285Asn	m	c.-119_-116delGTCA;						
c.940A>G	NA;p.Asn314Asp	spl	1	1%	1	1%			
c.392T>G	p.Phe131Cys	m	c.958G>A	p.Ala320Thr	m	1	1	1	1
c.563A>G	p.Gln188Arg	m	c.611G>C	p.Arg204Pro	m	1	1	1	1
c.329-2A>C	NA	spl	c.982C>T	p.Arg328Cys	m	1	1	1	1
c.329-2A>C	NA	spl	c.329-2A>C	NA	spl	1	1	1	1
c.563A>G	p.Gln188Arg	m	c.152G>A	p.Arg51Gln	m	1	1	1	1
c.563A>G	p.Gln188Arg	m	c.152G>T	p.Arg51Leu	m	1	1	1	1
c.563A>G	p.Gln188Arg	m	c.490C>T	p.Gln164Ter	PTC	1	1	1	1
c.329-2A>C	NA	m	c.505C>A	p.Gln169Lys	m	1	1	1	1
c.563A>G	p.Gln188Arg	m	unknown	unknown		1	1	1	1
c.134C>T	p.Ser45Leu	m	c.563A>G	p.Gln188Arg	m	1	1	1	1
c.404C>T	p.Ser135Leu	m	c.563A>G	p.Gln188Arg	m	1	1	1	1
c.499T>C	p.Trp167Arg	m	c.563A>G	p.Gln188Arg	m	1	1	1	1
c.507+2T>C	NA	m	c.563A>G	p.Gln188Arg	m	1	1	1	1
c.152G>T	p.Arg51Leu	m	c.563A>G	p.Gln188Arg	m	1	1	1	1
c.563A>G	p.Gln188Arg	m	c.812A>G	p.Glu271Gly	m	1	1	1	1
c.563A>G	p.Gln188Arg	m	1054G>C	p.Glu352Gln	m	1	1	1	1
c.425T>A	p.Met142Lys	m	c.584T>C	p.Leu195Pro	m	1	1	1	1
c.285T>G	p.Phe95Leu	m	c.855G>T	p.Lys285Asn	m	1	1	1	1
c.626A>C	p.Tyr209Ser	m	unknown	unknown		1	1	1	1
c.626A>C	p.Tyr209Ser	m	c.855G>T	p.Lys285Asn	m	1	1	1	1
c.812A>G	p.Glu271Gly	m	c.855G>T	p.Lys285Asn	m	1	1	1	1
c.584T>C	p.Leu195Pro	m	c.855G>T	p.Lys285Asn	m	1	1	1	1
c.626A>C	p.Tyr209Ser	m	c.855G>T	p.Lys285Asn	m	1	1	1	1
c.329-2A>C	NA	spl	c.748C>A	p.Pro250Thr	m	1	1	1	1
c.563A>G	p.Gln188Arg	m	c.499T>C	p.Trp167Arg	m	1	1	1	1

*NA* doesn't apply, *unknown* not detected, *m* missense, *spl* splicing, *PTC* premature termination codon

^*^Study limitation: the analytical method used does not exclude the possibility of the presence of a large deletion within the *GALT* gene in one allele in the case of homozygous patients

## Discussion

Among the commonest *GALT* variants described in HGMD, c.563A>G (p.Gln188Arg) and c.855G>T (p.Lys285Asn) are the most abundant in our Polish group, identified with the frequency of 49.4% and 32.2%, respectively. Other recurrent variants like c.329-2A>C (3.2%), c.997C>T (p.Arg333Trp; 1.7%), and c.626A>C (p.Tyr209Ser; 1.7%) are far less frequent, while the majority of remaining variants are rare and were detected in less than 1.5% of the alleles. In terms of p.Gln188Arg and p.Lys285Asn, the observed frequencies are similar to those published by our group in 2001 [13], where those mutations were identified in 54% and 28% of mutated alleles, respectively. According to other research, the p.Gln188Arg was reported in approximately 70% of the alleles affected with GALT deficiency from the population of northern Europe [14], which is much higher than in our cohort. Simultaneously, the p.Lys285Asn was more prevalent in central European countries, suggesting its Slavic origin [14]. Indeed, according to international multicenter data gathered from December 2014 to July 2018 among 404 genotyped patients, 233/404 (57.7%) were homozygous for p.Gln188Arg, 7/404 (1.7%) were homozygous for p.Lys285Asn, and 29/404 (7.2%) were heterozygous p.Gln188Arg/p.Lys285Asn [15]. On the contrary, in our group, 53/175 (30.3%) and 26/175 (14.8%) probands were homozygotes for p.Gln188Arg and p.Lys285Asn, respectively. The 39/175 (22.3%) were identified as harboring p.Gln188Arg *in trans* with p.Lys285Asn. The limitation of our study is that the DNA of the parents was not accessible, therefore, we cannot rule out that certain homozygous cases were in fact heterozygous for identified mutation and 5-kb deletion (precisely: NM_000155.2(*GALT*):c.[− 1039_753del;820+50_*789delinsGAATAGACCCCA]) spanning whole *GALT* gene. This deletion was first characterized by Coffee et al. Genet Med. 2006 Oct;8(10):635–40 in patients of Ashkenazi Jewish ancestry. The is no precise data on the frequency of this deletion in Europe. In multicenter data analysis of Rubio-Gozalbo et al. the deletion was reported as homozygous in 3/509 patients and in heterozygous state in 5/509 patients [15], while in the Mexican group the deletion was present in 3/27 patients [PMID: 23430845].

Regarding the rare variants, accounting for over 18% of *GALT* alleles in our study, we noticed that 20 out of 29 detected variants were identified in one or two cases, including two novels: p.Pro87Leu and p.Glu352Gln. This observation shows that the heterogeneity of these variants in Polish patients is much more significant than expected.

Although most classical cases manifest clinical symptoms soon after birth, in the proband from Family A (genotype p.Pro87Leu/p.Tyr209Ser), a rare asymptomatic course of GALAC1 in the neonatal period was observed. The first symptoms were observed after 4 weeks of breastfeeding, but galactosemia was finally diagnosed at the age of 5 months, and GALT activity was about 5% measured at that time. Interestingly, in the patient's younger sister, the GALT activity was about 1% on the first day of life.

In the literature, galactosemia with GALT activity between 1 and 10% is sometimes referred to as clinic variant galactosemia. The most typical variant causing this phenotype is p.Ser135Leu. Unfortunately, we do not have biochemical data on the mentioned variants among our patients. Most probands did not have a galactose-1-phosphate assay performed prior to starting the diet because, as a rule, the diet should be started as soon as galactosemia is suspected, before the diagnosis is confirmed. The gal-1-P assay is only performed at the Institute of Mother and Child, and most patients had it completed at the first visit to our department, being already on a diet. In the case of another novel variant—p.Glu352Gln, detected *in trans* with p.Gln188Arg, the clinical picture was typical for classic galactosemia with undetectable GALT activity.

The majority of probands in our cohort manifests symptomatic course of galactosemia (data not shown), which could be anticipated, considering the high percentage of patients harboring p.Gln188Arg and p.Lys285Asn variants, associated with very low (0.2% compared to wild type) or non-detectable erythrocyte GALT enzyme activity [16]*,* and the fact that they were diagnosed based on clinical symptoms.

Except for classic galactosemia discussed mainly herein, only three individuals have been diagnosed with Duarte galactosemia (biochemical variant galactosemia), resulting from a heterozygous c.[-119_-116delGTCA;c.940A>G] variant commonly referred to as Duarte 2 (D2 *GALT* variant), *in trans* with the pathogenic variant. Because the Duarte 2 allele's allelic frequency is 6.5% in the European non-Finnish general population (according to the GnomAD database, https://gnomad.broadinstitute.org), we conclude that this variant is highly underrepresented in our cohort. Consequently, since galactosemia is not among the diseases diagnosed through the national program of newborn screening in Poland, the vast majority of patients in this study were apparently all identified based on symptoms, not by population screening. We assume that some mild cases are not recognized at all what means there is ascertainment bias in the cohort, as infants who did not exhibit symptoms leading to diagnosis would have been missed. This may explain the under-representation of infants with Duarte galactosemia and may have also biased against finding some hypomorphic alleles that might not have caused acute neonatal disease. Interestingly, the Argentinian study published last year enabled the identification of Duarte-like variant p.Glu230Lys in 4% of alleles of galactosemia patients, identified by clinical symptoms or by positive NBS [17]. This clearly shows that the molecular spectrum of *GALT* gene variants is not yet fully discovered.

## Summary

To the best of our knowledge, the described cohort of 195 patients with galactosemia is the largest single-center cohort presented so far. Our results support the previous observations that the p.Lys285Asn variant originates from Central Europe. However, over 18% of *GALT* alleles are sporadic variants, including two novel ones, p.Pro87Leu and p.Glu352Gln. Our results broaden the knowledge on the molecular pathology and epidemiology of GALT deficiency.

## Supplementary Information

**Additional file 1. Table S1**: Primers used in diagnostic procedure

## Data Availability

The datasets used and/or analyzed during the current study are available from the corresponding author on reasonable request.

## Abbreviation

GALT: Galactose-1-phosphate uridylyltransferase
