# Supplementary material for: The genetic basis of classical galactosaemia in Polish patients
Source: Orphanet J Rare Dis. 2021 May 24;16:239. doi: 10.1186/s13023-021-01869-3 (PMC8142503; doi:10.1186/s13023-021-01869-3)
Supplement: Supplementary file 1 — Additional file 1. Table S1: Primers used in diagnostic procedure [file 13023_2021_1869_MOESM1_ESM.docx]

| exons | forward primer name | forward primer sequence | reverse primer name | reverse primer sequence |
| --- | --- | --- | --- | --- |
| 1-3 | GALT_1F | GTGGCTCTAGCTCTGGGTGA | GALT_3R | CCCARTGCTGAGTCTCCAAC |
| 4-5 | GALT_4FN | GTATGGGGCAGTGAGTGCTT | GALT_5RN | TCATGGCACCTTTGTTTTCA |
| 6-7 | GALT6i7F | AGGGGGTGATGAAGCTTTG | GALT6i7R | TGCTAAGGCCTCCTAGCAAGT |
| 8-9 | GALT_8F | GAGGTGGBGAGAAGACATCA | GALT_9R | ACTTCCCCCAGCAACTGTTT |
| 10-11 | GALT_10F | GGTTGGGTTTGGGAGTAGGT | GALT_11R | TGGGGTAGATTTTTGCATCA |

Table S1 Primers used in diagnostic procedure

The primers were optimised not to contain variants with MAF >0,01%.
